# Global Bibliometric and Phylogenetic Analysis of mcr‐Mediated Colistin Resistance

**DOI:** 10.1155/bmri/8343626

**Published:** 2026-07-20

**Authors:** Md Zulfekar Ali, Uwem Okon Edet, Umego Chukwdi Theodore, Francisca Nwaokorie, Md Hafizur Rahman, Edrous Alamer, Hazem Mathkour, Clement Meseko

**Affiliations:** ^1^ Animal Health Research Division, Bangladesh Livestock Research Institute, Savar, Dhaka, Bangladesh, blri.gov.bd; ^2^ Regional Laboratory for Animal Influenza and Transboundary Animal Diseases, National Veterinary Research Institute (NVRI), Nigeria, nvri.gov.ng; ^3^ Department of Microbiology, School of Pure and Applied Sciences, Federal University of Technology, Akwa Ibom State, Nigeria, futa.edu.ng; ^4^ Department of Biology Education, School of Science, Federal College of Education (Technical), Umunze, Anambra State, Nigeria, fcetakoka-edu.net; ^5^ Department of Medical Laboratory, College of Medicine, University of Lagos, Lagos, Nigeria, unilag.edu.ng; ^6^ Department of Medical Laboratory Technology, Faculty of Nursing and Health Sciences, Jazan University, Jazan, Saudi Arabia, jazanu.edu.sa

**Keywords:** bibliometric, colistin, gaps, *mcr gene variants*, One Health, phylogenetic analysis, plasmid

## Abstract

The emergence and spread of mobilized colistin resistance (*mcr*) genes threaten the efficacy of colistin, a last‐resort antibiotic used in the management of multidrug‐resistant Gram‐negative bacteria (GNB). This study was aimed at (1) providing a bibliometric assessment of the mcr‐related research landscape and (2) conducting phylogenetic and metadata analyses of all known mcr gene variants to elucidate their relatedness and global distribution by country of origin, sample type, and harboring bacterial diversity. Bibliometric analysis was conducted following the SPAR‐4‐SLR methodology, where *mcr*‐related publications were retrieved from Scopus and analyzed using diverse packages in R software, Datawrapper, the VOSviewer tool, and Biblioshiny. Genomic analysis was conducted on 116 complete *mcr* gene sequences retrieved from the National Center for Biotechnology Information (NCBI). A neighbor‐joining phylogenetic tree was constructed to infer evolutionary relationships, and the associated metadata of sequences were analyzed. A total of 3936 mcr‐related publications met the inclusion criteria. Since the discovery of *mcr* in 2015, research output has grown consistently through 2024, reflecting an intense global scientific mobilization and the rapid prioritization of colistin resistance given its significant public health threat. About 54.15% of studies appeared in Top Q1–Q2 journals, notably *Frontiers in Microbiology* and *Antimicrobial Agents and Chemotherapy*. The most cited work was Liu et al. (2016), with 4445 citations. China and India led in publications and collaborations, while China dominated funding. Phylogenetic analysis revealed 10 *mcr* variants, each clustering together. Distance analysis showed that *Escherichia coli* was the likely origin of *mcr*‐1–2 and *mcr*‐5 variants, *Aeromonas* of *mcr*‐3, *Moraxella* of *mcr*‐6, *Klebsiella pneumoniae* of *mcr*‐7, *Raoultella ornithinolytica* of *mcr*‐8, and *Enterobacter cloacae* of *mcr*‐10. However, *mcr*‐4 and *mcr*‐9 showed multiple origins with similar branch lengths for *E. coli*, *Salmonella*, and *Enterobacter*. The widespread occurrence of *mcr*‐1–10 across diverse bacterial species underscores the urgent need for an integrated, One Health surveillance approach to monitor and mitigate colistin resistance spread globally.

## 1. Introduction

Antimicrobial resistance (AMR) is one of the pressing public health challenges of the 21^st^ century, particularly among bacteria [1, 2]. AMR is pervasive and has become a significant global public health issue [3]. This aligns with earlier reports that also showed the presence of AMR across diverse microbial species, including *Escherichia coli* and *Staphylococcus aureus* in Africa from environmental and clinical settings [4, 5]. No known drug in clinical use, including last‐resort antibiotics, is spared the resistance menace. Colistin, once the ultimate defense against multidrug‐resistant (MDR) GNB, has now been increasingly compromised by the emergence and spread of mobilized colistin resistance (*mcr*) genes [6]. Initially discontinued due to toxicity concerns, colistin regained clinical prominence as resistance to other antibiotics surged [7]. However, the global dissemination of *mcr* genes has severely undermined its clinical value [8].

The emergence of *mcr* genes among GNB around the globe illustrates the complexity and reach of the AMR crisis [9]. While several scholars advocate for prudent antibiotic use and the development of new antimicrobial agents [6], others emphasize a multidisciplinary, One Health–based strategy [8]. Since its discovery in China in 2015, *mcr* has evolved into 10 variants (*mcr*‐1 to *mcr*‐10), each with distinct genetic and ecological characteristics [8, 10]. These plasmid‐mediated genes encode phosphoethanolamine transferases that modify lipid A, reducing colistin affinity and enabling horizontal gene transfer across bacterial species and geographic boundaries [8]. The gene has a global spread across diverse sample types and spans several countries [10]. Their co‐occurrence with other resistance genes, such as *bla*_KPC_ and *bla*_NDM_, further limits treatment options [7].

Several bibliometric analyses exist that have examined diverse aspects of colistin resistance around the world [11–13]. Nwabor et al. [12] examined colistin resistance in *Klebsiella pneumoniae* between 1995 and 2019 globally, leaving out other pathogens that have been shown to elaborate resistance to colistin. Yakubu et al. [13] conducted a bibliometric analysis between 1973 and 2019 aimed at an understanding of the global trends and status in colistin resistance research. The time span of their study leaves out a synthesis of the research landscape for 2020–2025. On the other hand, Gördük and Kelle [11] examined colistin‐related theses in Türkiye, leaving out other countries in their studies. As indicated by the scope of these studies, the colistin resistance research landscape is fragmented, limited in scope, and does not capture the global picture.

Recent studies have expanded our molecular and phylogenetic insights into *mcr* variants [7], whereas others have shown an increased adoption of the One Health framework in AMR since 2015 [14]. Variants *mcr*‐1, *mcr*‐2, and *mcr*‐6 likely originated from *Moraxella* spp., while *mcr*‐3 and *mcr*‐7 are associated with *Aeromonas* spp., suggesting multiple evolutionary pathways [10]. It is also known that clustering patterns reflect both sequence homology and geographic distribution [10]. Little is known about the origins of other *mcr* variants. Also, not fully understood are the sources and their roles in the spread of the various *mcr* genes among various isolates. Therefore, despite the growing body of literature on *mcr* genes, a comprehensive synthesis of global research data is lacking.

To fill these gaps, we conducted a combined bibliometric, metadata, and phylogenetic analysis of *mcr* genes globally. Bibliometric analysis systematically examines literature in a particular field with the aim of providing both quantitative and qualitative insights. As a study type, it has been applied to the study of avian influenza virus globally, HIV in Nigeria, and vaccine uptake in Africa [15–17]. Conducting a bibliometric analysis of *mcr* genes can reveal how the research landscape has changed over time, the evolution of keywords and themes, and the most prolific contributors (authors, institutions, and funders). Phylogenetic analysis, on the other hand, aims to provide insight into the genetic diversity, relatedness, and potential origins of *mcr* genes. Furthermore, by mapping the metadata to the genetic analysis, our study is aimed at further providing insight into the diverse bacteria that can harbor *mcr* genes and their sources. Our objectives are twofold: (1) to conduct a bibliometric analysis of the *mcr* research landscape globally to reveal the thematic evolution of keywords and study trajectory and (2) to analyze the metadata and phylogenetic relatedness of the *mcr* genes to reveal global distribution in terms of countries, years, sample types, and harboring bacteria diversity.

## 2. Methodology

### 2.1. Search Terms and Rationale

To obtain relevant studies for the bibliometric analysis, we searched the Scopus database comprehensively using the keywords detailed below: TITLE‐ABS‐KEY (“*mcr*” OR “*mcr*‐1” OR “*mcr*1” OR “*mcr*‐2” OR “*mcr*2” OR “*mcr*‐3” OR “*mcr*3” OR “*mcr*‐4” OR “*mcr*4” OR “*mcr*‐5” OR “*mcr*5” OR “*mcr*‐6” OR “*mcr*6” OR “*mcr*‐7” OR “*mcr*7” OR “*mcr*‐8” OR “*mcr*8” OR “*mcr*‐9” OR “*mcr*9” OR “*mcr*‐10” OR “*mcr*10” OR “mobile colistin resistance” OR “mobilized colistin resistance” OR (“polymyxin∗” OR “colistin”) AND “resistance” AND (plasmid OR transferable OR “mobile”)) AND PUBYEAR <2026 AND PUBYEAR >2014 AND PUBYEAR <2026 AND (LIMIT‐TO (LANGUAGE, “English”)) AND (LIMIT‐TO (PUBSTAGE, “final”)) AND (LIMIT‐TO (DOCTYPE, “ar”) OR LIMIT‐TO (DOCTYPE, “le”)). To ensure a comprehensive and targeted retrieval of literature on mobile colistin resistance, we designed a search strategy that captures both specific and broader terminologies relevant to the topic (as shown above). Scopus was selected due to its broad multidisciplinary coverage and inclusion of a large number of peer‐reviewed journals compared to more specialized databases like PubMed, which focus strictly on biomedical and medical literature [18, 19]. As a database, it has been utilized in a recent bibliometric study [20].

### 2.2. The Search Process

The bibliometric search was conducted in four stages. Initially, 5235 documents were retrieved from titles, abstracts, and keywords without a limit on the year of study. Refining to English‐language articles and reviews at the final publication stage reduced this to 4664 studies. Applying a 2015–2025 year filter yielded 4021 documents. After manual screening to remove duplicates and irrelevant studies, 3936 documents were finally included for analysis. Two groups of authors working independently screened the records. Any discrepancies regarding the inclusion or exclusion of studies were resolved through a consensus‐based discussion, adhering to standard systematic review guidelines [21].

### 2.3. Description of the Scientific Procedures and Rationales for Systematic Literature Reviews (SPAR‐4‐SLR) Protocol Adopted in the Study

**Figure 1:**
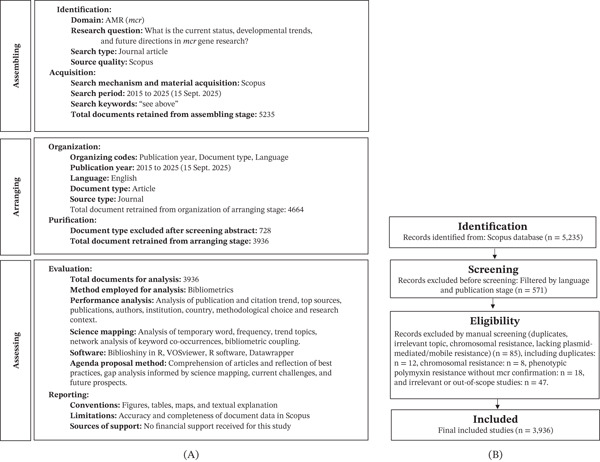
(A) Flowcharts showing the SPAR‐4‐SLR protocol (assembling, arranging, and assessing stages) and (B) PRISMA flow diagram showing the various steps utilized in the screening of the retrieved studies.

The methodology was guided by the SPAR‐4‐SLR framework [22]. Applying this framework ensured a structured, transparent, and reproducible approach to the literature review, enhancing the methodological rigor of the study (Figure 1). The literature screening process followed the SPAR‐4‐SLR framework to ensure systematic rigor in bibliometric data collection. The flow of study selection, mapped according to Preferred Reporting Items for Systematic Reviews and Meta‐Analyses (PRISMA) reporting standards, is also illustrated in Figure 1.

### 2.4. Bibliometric Analysis

Using R (Version 4.5.3) running in RStudio (Version 2025.09.0) [23], Datawrapper (https://www.datawrapper.de/) [24], VOSviewer Windows version 1.65 [25], and Biblioshiny Version 4.1 [26], the final bibliometric dataset was analyzed. Specifically, in Biblioshiny, the research trend in the last decade, cumulative keyword trends, thematic mapping, and factorial analysis were plotted. Keyword network and overlay visualizations were conducted using VOSviewer. Trajectory of mcr research globally, keyword and metadata analyses were done using RStudio. Datawrapper was used to visualize global trends, whereas collaborative networks among lead authors were illustrated using a chord diagram generated in RStudio.

### 2.5. Phylogenetic and Metadata Analysis

To infer the evolutionary pattern of the *mcr* variants and their transmission dynamics, we constructed a global phylogenetic tree and also analyzed their metadata. A total of 116 nonredundant nucleotide sequences were systematically retrieved from the National Center for Biotechnology Information (NCBI) GenBank database, targeting only NCBI Reference Sequences (RefSeq) for all 10 major mcr groups (*mcr*‐1 to *mcr*‐10) and their recognized subvariants (e.g., *mcr*‐1.1 and *mcr*‐1.2). As shown in Supporting Information 1: Table S1, a total of 116 *mcr* sequences and their associated metadata were included in this study. For each retrieved sequence, the recorded metadata included the microorganism′s name, host animal, country of origin, and *mcr* variants (see first column). The sequences were aligned in MAFFT v7.511 [27], applying the FFT‐NS‐2 model, and the resulting alignment file was used for the phylogenetic analysis, where the Jukes–Cantor substitution model with 1000 bootstrap replicates was applied. The resulting *Newick* tree was visualized using Interactive Tree Of Life (iTOL) v5 [28].

## 3. Results

### 3.1. Bibliometric Analysis

#### 3.1.1. Study Trajectory Since 2015 and Their Yearly Citations

**Figure 2:**
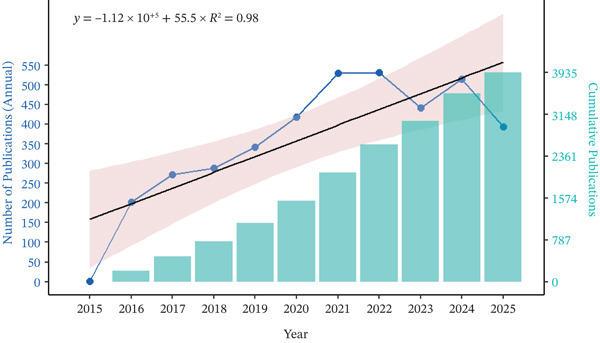
Trajectory of research studies on *mcr* genes, 2015–2025, using a combined bar chart and scatter plot. The scatter plot illustrates the annual number of publications (blue dots, left *y*‐axis) and the cumulative number of publications (teal bars, right *y*‐axis).

A total of 3936 studies were included in this bibliometric analysis. The *mcr*‐related research began in 2015 with a single publication, rising sharply to 203 in 2016. This upward trajectory was maintained, reaching a peak of 531 documents in 2022. Slight declines occurred in 2023 and 2025, with 441 and 394 publications, respectively, while 2024 saw a rebound to 515 studies. Our equation of trend is *y* = −1.12 × 10^+5^ + 55.5×, whereas the correlation coefficient is *R* ^2^ = 0.98 (Figure 2). Supporting Information 2: Table S2 shows the number of publications and their yearly citations over the last decade (2015–2025). The number of publications increased exponentially between 2015 and 2022, rising from 1 to 531, but declined from 2023 onward. In contrast, the number of citations steadily decreased from 2016 to 2025, ranging from 17,484 to 331. Also captured is the average citation for each year, and the result indicates a decrease from 2015 to 2025, with values that ranged from 2 to 331. Supporting Information 2: Table S2 presents the mean total citations per year, showing a trend from 30.09 in 2015 to 0.54 in 2025. This downward trajectory aligns with the natural citation accumulation life cycle, where more recent studies have had fewer years to accrue citations compared to earlier publications. Furthermore, as shown in Figure 2 and Supporting Information 2: Table S2, the total number of publications peaked in 2022 (531), while citations peaked in 2016 (17,484 for 203 documents) and have since declined exponentially, reaching 2019 in 2024.

#### 3.1.2. Journal Destinations for the Top 30 Articles on *mcr* and Most Cited Articles

Supporting Information 3: Table S3 summarizes the Top 30 journals publishing mcr studies, highlighting their percentage contribution to the field, total citations, impact factors (IFs), and quartile rankings. Together, the Top 30 journals published a total of 2525 articles (54.15% of all publications). Most journals were Q1 (*n* = 10) or Q2 (*n* = 17) with IFs, except *Microbial Drug Resistance*, *Journal of Medical Microbiology*, and *Veterinary Microbiology*, which are yet to be assigned IFs. *Frontiers in Microbiology* led in publications (350 articles), while *Antimicrobial Agents and Chemotherapy* had the highest citations (8563). The journal landscape included core, specialized, and multidisciplinary outlets. Supporting Information 4: Table S4 summarizes the Top 20 most cited papers in *mcr* research, including authors, digital object identifiers (DOIs), total citations, and citations per year. The Top 3 articles were led by Liu and published in *The Lancet* (2016), Xavier in *Eurosurveillance* (2016), and Poirel in *Microbiology Spectrum* (2017). These pioneering studies accrued 4445, 673, and 643 total citations, with average citations per year of 445, 67, and 80, respectively. The key findings of these studies have been synthesized, and they are captured in the Discussion section.

#### 3.1.3. Most Prolific Authors and Their Affiliations and Collaboration Network

**Figure 3:**
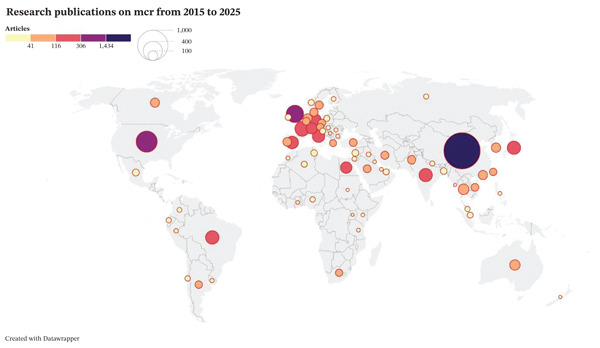
The research publications on *mcr* across the globe. Color legends indicate the number of publication points received from 2015 to 2025.

**Figure 4:**
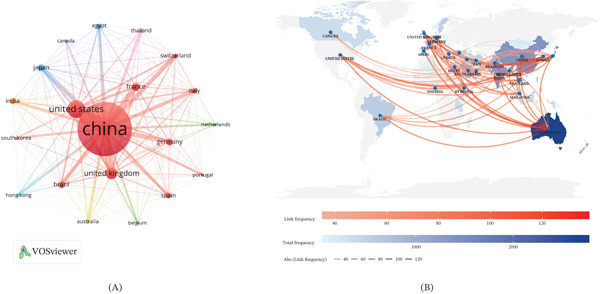
(A) Bibliometric coupling network of authors affiliated with the Top 20 countries by publication output (VOSviewer visualization). (B) Geographical visualization of the international research collaboration network. The map displays research collaboration links between countries, where the color and thickness of the curved lines represent the frequency of collaborations.

Supporting Information 5: Table S5 summarizes authors with at least 31 publications on mcr‐related studies, capturing their rankings (1–17), author names, publication counts, citation totals, institutional affiliations, and countries of affiliation. The results indicate that 17 authors met this criterion. The author Ruichao Li was the most prolific with 77 articles, followed by Wang Y. with 62, whereas all other authors had ≥ 31 publications. Wang Y. and Zhang R. received the highest citations, followed by Patrice N. Affiliations spanned multiple countries, dominated by China (*n* = 11), followed by Switzerland (*n* = 2), Hong Kong (*n* = 2), and France (*n* = 1) (Figure 3). South China Agricultural University contributed nine prolific authors, with similar single‐institution dominance seen in Switzerland and Hong Kong. Top publishers were mainly from China (1434 publications) and the United States (466) between 2015 and 2025. Bibliometric coupling networks (Figure 4A,B) show a hierarchical global research landscape, with China and the United States emerging as the primary collaborative nodes. China leads in both productivity and connectivity, with a total link strength (TLS) of 491,860 (Supporting Information 6: Table S6). While publication volume generally correlates with collaborative connectivity, countries such as Brazil (TLS: 74,337) and India (TLS: 53,203) demonstrate disproportionately high connectivity relative to their document counts, highlighting their function as critical conduits for international knowledge exchange. Africa had minimal representation.

#### 3.1.4. Most Prolific Funders of *mcr* Research Globally

The Top 10 most prolific funders of *mcr* research globally and their countries are presented in Supporting Information 7: Table S7. The table shows funder rankings (1–10), funder names, countries, and the number and percentage of supported documents. The result indicates that Chinese funders dominate the Top 4 funders. The top most funder, along with 835 documents, was the National Natural Science Foundation of China and was followed by the National Key Research and Development Program of China with 299 documents. Funders from the United States and Brazil were well represented with two funders each. Apart from the European Union (EU), other funding sources from the EU region made the list of Top 10 funders.

#### 3.1.5. Keyword Analyses

**Figure 5:**
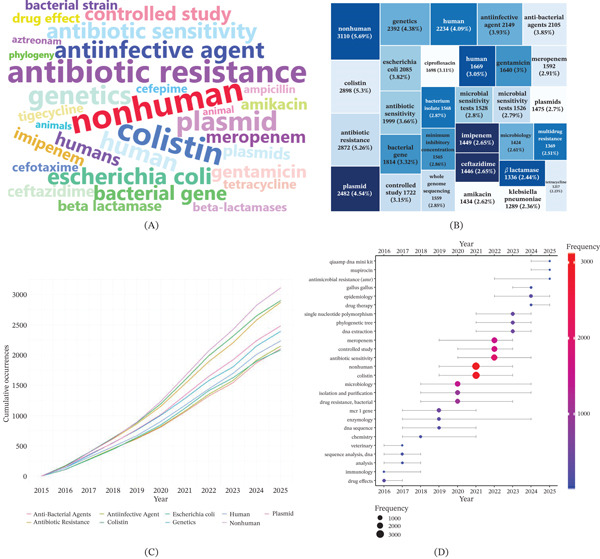
Various aspects of the analyzed keywords on *mcr* studies. (A) Word cloud showing the diverse keywords in terms of occurrence, with font sizes depicting their frequency of usage. (B) Word title illustration of the keywords showing their percentages. (C, D) The usage of the keywords over time and their cumulative usage over time.

Keyword analysis in Figure 5A–D highlights the diversity, frequency, and temporal evolution of terms associated with *mcr* research. Figure 5A shows a wide range of keywords with *nonhuman*, *colistin*, *antibiotic resistance*, and *plasmid* appearing most frequently. Figure 5B quantifies these terms, displaying their counts and percentages in descending order. Temporal trends in Figures 5C–D reveal how keyword usage evolved from 2016 to 2024. The earliest terms, *drug effect* and *immunology*, appeared in 2016, whereas *enzymology* was consistently used from 2017 to 2024. The keyword *mcr-1 gene* showed concentrated use between 2017 and 2021. From 2022 onward, newer terms emerged, including *drug therapy* and *epidemiology*.

### 3.2. Meta‐Analyses and Phylogenetic Analysis of *mcr*‐1–10

**Figure 6:**
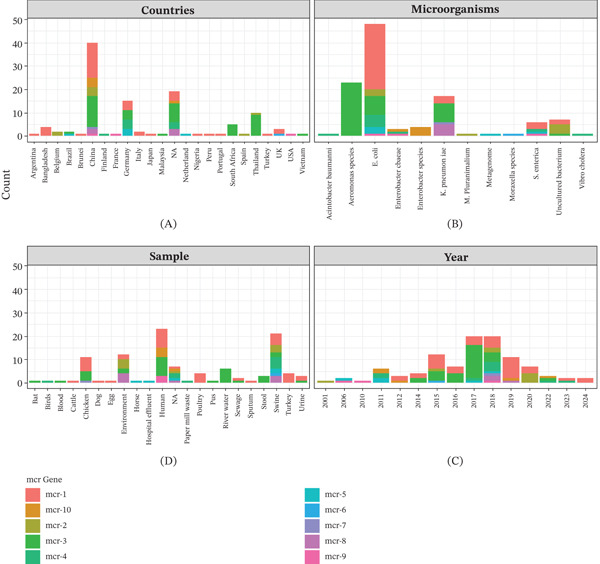
A facet plot summarizing *mcr* gene distribution (*mcr*‐1 to *mcr*‐10) across diverse categories. (A) The country‐wise distribution, (B) the distribution among microbial taxa, (C) the year‐wise distribution, and (D) the distribution across different sample sources.

Figure 6A–D presents the global distribution of *mcr* variants across countries, microorganisms, sample types, and years. Figure 6A shows that Brazil, China, Germany, and the United Kingdom each reported at least one *mcr* gene, with China and Germany hosting the widest diversity. China contained *mcr*‐1 to *mcr*‐3 and *mcr*‐7 to *mcr*‐10 across multiple sample types, whereas Germany reported *mcr*‐1 and *mcr*‐3 to *mcr*‐5. Among all variants, *mcr*‐1 showed the broadest international spread, occurring in Portugal, the United Kingdom, China, Bangladesh, Nigeria, Germany, Italy, Japan, Turkey, Brunei, Argentina, and Peru. In contrast, *mcr*‐6 and *mcr*‐7 were each detected in only one country. Microbial distribution (Figure 6B) revealed 12 host organisms. *E. coli* was the predominant carrier, especially for *mcr*‐1 (*n* = 28), followed by *Aeromonas* spp. with *mcr*‐3 (*n* = 23) and *K. pneumoniae* with *mcr*‐3 and *mcr*‐8. *Salmonella enterica* carried multiple variants, including *mcr*‐1, *mcr*‐4, *mcr*‐5, and *mcr*‐9, whereas other organisms such as *Vibrio cholerae*, *Moraxella*, *Acinetobacter*, and *Enterobacter* species showed limited but diverse representation. Figure 6C shows that *mcr* genes appeared across multiple years from 2001 to 2024. *mcr*‐1 had the widest temporal distribution, whereas the earliest variant was *mcr*‐2, detected in 2001. *mcr*‐6 and *mcr*‐7 showed the lowest abundance, appearing only in 2015 and 2018.

Figure 6D shows the global diversity of sample sources for *mcr* genes, with *mcr*‐1, *mcr*‐3, and *mcr*‐4 appearing across the widest range of sample types. Human and swine samples were the most common, highlighting clinical and livestock environments as key reservoirs. *mcr*‐1 showed the broadest spread, occurring in humans, livestock, poultry, and environmental samples. Environmental matrices such as river water, sewage, and effluents also frequently contained *mcr*‐3 and *mcr*‐8.

**Figure 7:**
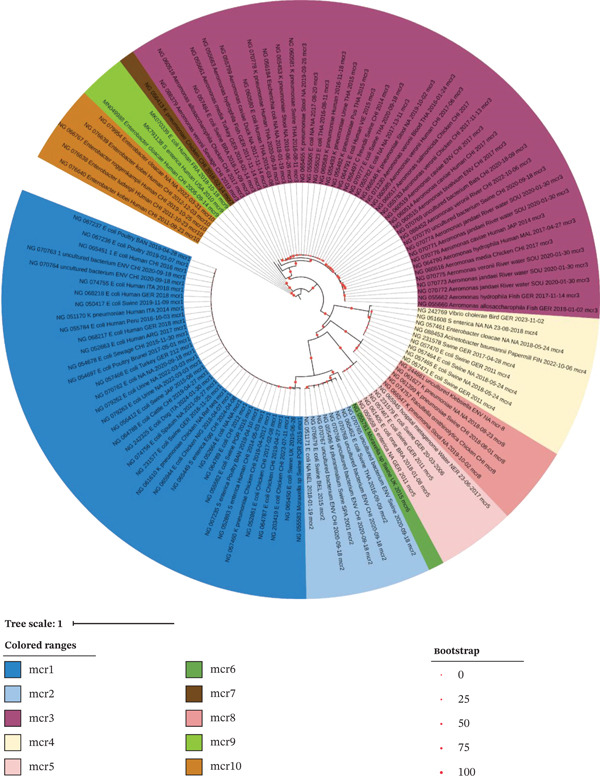
Phylogenetic analysis of the various *mcr* variants (1–10) isolated (*n* = 116) across the globe, constructed using a neighbor‐joining (NJ) tree and visualized in iTOL tree. Legend = assigned bootstraps and variants (top and bottom).

Figure 7 presents a circular neighbor‐joining tree illustrating the phylogenetic relationships among the *mcr* gene variants. The analysis shows clear and distinct clustering, with each variant grouping tightly with its own sequences. *mcr*‐1 and *mcr*‐3 were the most abundant variants, while *mcr*‐6 and *mcr*‐7 appeared as single‐sequence lineages. *mcr*‐3 formed the second‐largest clade and was strongly associated with *Aeromonas* species from aquatic environments in China and Germany. Smaller but well‐defined clusters of *mcr*‐4 and *mcr*‐5 involved *Enterobacter cloacae*, *E. coli*, and *V. cholerae* from environmental and animal sources. Variants *mcr*‐8, *mcr*‐9, and *mcr*‐10 formed distinct clusters linked to *Raoultella ornithinolytica*, *E. coli*, and *E. cloacae* species, mainly from clinical settings in Europe and Asia. Variants *mcr*‐9 and *mcr*‐10 occupied basal positions in the NJ tree.

## 4. Discussion

The study combined bibliometric and phylogenetic analyses of genes of *mcr* variants and their metadata with the aim of providing a holistic insight into the research landscape and the spread of the *mcr* genes globally. The first study on *mcr* was reported in 2016, and following its publication, there has been an astronomical increase in studies on *mcr* globally, reaching 3936 studies without a notable phase of slow growth. This is in contrast to other bibliometric studies that showed a period of slow growth that is usually followed by a period of increased studies [16, 17]. This trajectory could be a result of established protocols for the study of AMR compared to other areas of microbiology. Our coefficient of correlation showed a strong positive correlation between the number of studies and the year, indicating that other factors are less likely to be responsible for driving *mcr* publications. Collectively, the increase in the number of publications could be due to increased interest from scientists conducting studies on *mcr* variants, sparked by the fact that colistin, a last‐line antibiotic for the management of GNB, is now experiencing resistance at an alarming rate [6, 8].

Our analysis of the top journal destinations for the studies showed a preference by the authors for a few journals that published 54% of the total studies, and this aligns with a previous study [29]. These journal outlets covered specialized, multidisciplinary, and area‐specific journals. This spread of journals aligns with previous studies [16, 17], with the majority within Q1 and Q2 rankings with IFs, indicating high visibility and academic credibility [29]. The most cited study by Liu et al. [30] led with 4445 citations, showing the importance of pioneering studies in any field, and aligns with previous bibliometric reports that showed high citation for pioneer studies in their fields [6, 8]. The study by Liu et al. [30] heralded the *mcr* resistance era. Since then, the discovery and characterization of mobile colistin resistance (*mcr*) genes have unfolded rapidly. All the Top 20 studies provided distinct insights into various aspects of *mcr*.

Hasman et al. [31] first detected *mcr*‐1 in Danish patients and imported chicken meat, indicating zoonotic and food‐borne transmission. Subsequent reports in 2016 expanded our understanding with the report of *mcr*‐2 in pigs in Belgium [32], whereas McGann et al. [33] in 2016 reported the first US case of *E. coli* carrying both *mcr*‐1 and blaCTX‐M. Mechanistic insights also emerged from studies by Baron et al. [34] in 2016. In the same year, 2016, Rolain et al. [35] detected *mcr*‐1 genes in clinical samples. In 2017, *mcr*‐3 was identified in *E. coli* [36], *mcr*‐4 in *Salmonella* and *E. coli* across Europe [37], and *mcr*‐5 was linked to a transposon in *Salmonella* Paratyphi B [38]. Jeannot et al. [39] further explored polymyxin resistance pathways. The following year, *mcr*‐7.1 was detected in *K. pneumoniae* [40] and *mcr*‐8 in animals and humans, often co‐occurring with blaNDM [41], whereas Poirel et al. [42] reviewed MDR in *E. coli*. Rebelo et al. [43] developed a multiplex PCR assay to detect *mcr*‐1 to *mcr*‐5. Wang et al. [41] reported the widespread distribution of *mcr*‐1 across continents and plasmid types. In 2019, *mcr*‐9 was detected in colistin‐susceptible *Salmonella typhimurium* [44], suggesting silent dissemination. The most recent addition, *mcr*‐10, was identified as a novel plasmid‐borne gene [45], underscoring the ongoing evolution of mobile colistin resistance.

Our authorship, as well as their affiliations and funders′ analyses, indicates that Ruichao Li and Wang Y. lead global *mcr*‐related research output, with China emerging as the dominant contributor. Other significant contributors included India, the United States, Brazil, and European nations, whereas Africa remained under‐represented. Funding analysis highlights the National Natural Science Foundation of China and other Chinese agencies as major funders of *mcr* research, reinforcing China′s central role in advancing global AMR surveillance. Collaborative network analysis showed the dominance of China, Australia, the United States, and income‐rich countries in Europe. Notably missing were collaborative networks from Africa, despite bearing a significant brunt of the AMR menace. This paucity of collaboration and prolific authors aligns with previous studies that showed a dominance of income‐rich countries over resource‐poor settings [16, 17]. These disparities can be explained by several factors, including limited research infrastructure and access to internal and external funding in income‐poor nations compared to richer Western countries. Others include poor emphasis on AMR and a lack of a national action plan to guide AMR research as a whole from a One Health perspective. The specific lack of genomic surveillance infrastructure specifically for mcr gene studies in Africa limits its detection in the region and also has a direct public health implication, especially given the clinical importance of colistin resistance. A stronger call for enhanced capacity building and funding targeted at mcr and AMR as a whole is urgently needed to bridge the observed infrastructure and funding divide.

Furthermore, to strengthen the drive from a One Health perspective, there is a need for a robust, more integrated, and actionable surveillance framework for *mcr* genes. Specifically, setting up a coordinated surveillance system that links every arm of the One Health framework, including clinical, veterinary, and environmental data, to enable real‐time tracking of *mcr* dissemination is urgently needed. To achieve this, a systematic collection and integration of livestock colistin usage data, particularly in poultry and cattle production, with routine screening of clinical isolates from hospitals and environmental monitoring of wildlife, bats, birds, hospital effluent and wastewater, surface water, and agricultural soils needs to be set up. Also, there is a need for creating genomic surveillance platforms to specifically provide whole‐genome sequencing data of isolates across all sectors, allowing for the identification of transmission pathways and plasmid‐mediated gene flow patterns. Given the inconsistent metadata reporting observed, there is a need for standardized metadata reporting, including specifically described sample source, antimicrobial exposure history, and geographic location, to allow for effective comparability across regions. Furthermore, setting up national and regional AMR databases that combine *mcr*‐specific indicators, together with strengthened laboratory capacity in under‐represented regions such as Africa, is critical.

Our analysis of keywords revealed some interesting findings. Keyword analysis revealed an evolving focus of *mcr*‐related research, with early studies (2016–2018) emphasizing mechanistic aspects such as drug effects, immunology, and enzymology, while later years (2020–2024) shifted toward epidemiological and applied themes like *Gallus gallus*, drug therapy, and plasmid‐mediated resistance. The dominance of terms such as “colistin,” “nonhuman,” and “antibiotic resistance” reflects sustained interest in resistance mechanisms and zoonotic pathways. However, there is limited exploration of environmental reservoirs and evolutionary adaptation under diverse selective pressures. Also, urgent studies are needed to integrate metagenomic, ecological, and One Health approaches.

The meta‐analysis and phylogenetic assessment of *mcr*‐1 to *mcr*‐10 genes provide further crucial insights. Analyzed metadata showed that China and Germany are major hotspots, consistent with previous findings [46]. China exhibited the highest diversity, containing *mcr*‐1 to *mcr*‐3 and *mcr*‐7 to *mcr*‐10 across human, animal, and environmental samples. Germany similarly displayed broad representation, especially *mcr*‐1 and *mcr*‐3 to *mcr*‐5, underscoring its role in the spread of colistin resistance. Among all variants, *mcr*‐1 showed the widest global presence, detected across Europe, Asia, Africa, and South America, aligning with earlier reports, highlighting its extensive dissemination 10, 47. Although the first published report appeared in 2016 [30], the earliest detected variant was *mcr*‐2 from *Moraxella pluranimalium* collected in 2001 in Spain, suggesting that *mcr* genes existed long before their clinical recognition. *mcr*‐1 demonstrated the broadest temporal spread, appearing consistently from 2012 onward, consistent with claims that it has circulated in the Chinese food chain since the 1980s [48]. Conversely, *mcr*‐6 and *mcr*‐7 appeared sporadically between 2015 and 2018 and remain rare, possibly due to limited dissemination or undersampling.

The bibliometric data reveals a marked shift toward the connection of *mcr* genes and carbapenem resistance—the most persistent clinical threat posed by *mcr*. The co‐occurrence of mcr with carbapenemases (e.g., *bla*_KPC_ and *bla*_NDM_) produces a pan‐drug‐resistant phenotype, effectively neutralizing last‐line therapeutic options [13, 49]. Our thematic analysis captures this evolution; however, early literature (2015–2018) focused on gene discovery, whereas recent research (2020–2025) increasingly addresses these high risks, including coresistant clones. This evolution signals a growing clinical urgency, as these superbug combinations fundamentally complicate treatment and raise mortality risks in hospital settings.

Sample diversity patterns indicate that *mcr*‐1, *mcr*‐3, and *mcr*‐4 are the most widely distributed across sample types, reflecting strong ecological adaptability and aligning with reports of *mcr*‐1 in global ecosystems [50, 51]. Predominantly found in human and swine samples, clinical and livestock environments serve as major reservoirs, likely due to extensive colistin use [52]. The various variants were detected in various environmental (hospital effluent, stool, and paper mill effluent), clinical (blood, sputum, and urine), and animal (bats, birds, cattle, dog, and poultry) samples. Their detection in these diverse environments aligns with previous reports examining various isolation sources [47, 48, 53] and the changing epidemiology of *mcr* [10]. Their detection in these diverse environments underscores their zoonotic and environmental cycling potential [10, 47, 48]. Specifically, it indicates that there is an active recycling of the mcr‐resistant pathogens between the human, animal, and environmental interfaces, with the potential to seed clinical settings, further compounding clinical outcomes. This aligns with a previous report that showed a link between mcr‐resistant *E. coli* and products and samples from aquaculture practices in China to humans [53]. Furthermore, the detection of variants *mcr*‐3 and *mcr*‐8 in aquatic systems highlights water′s role in dissemination. Flies may act as vectors linking environmental and host‐associated reservoirs [54]. Among the keywords used in the various studies, sample types as they relate to animals and environmental ecosystems are grossly under‐represented compared to humans and nonhumans, which were among the Top 20 keywords. However, the higher abundance of nonhuman‐based studies loosely indicates that studies examining the potential roles of the environment in the spread of mcr‐resistant variants are on the increase globally. *E. coli* dominates as a host, particularly for *mcr*‐1, followed by *Aeromonas* spp. and *K. pneumoniae*, consistent with Enterobacteriaceae prevalence [55]. Detection in *Salmonella*, *Enterobacter*, *Vibrio*, *Acinetobacter*, and *Moraxella* demonstrates the broad host range of *mcr* genes.

Phylogenetic analysis revealed distinct clustering patterns among the *mcr* variants, demonstrating their largely independent evolutionary trajectories. *mcr*‐1 and *mcr*‐3 formed the largest and most diverse clades, with *mcr*‐3 showing strong dominance in *Aeromonas* species from aquatic environments, consistent with previous reports [50, 51]. Smaller but well‐supported clusters of *mcr*‐4 and *mcr*‐5 occurred in *E. cloacae*, *E. coli*, and *V. cholerae*. Variants *mcr*‐8, *mcr*‐9, and *mcr*‐10 were closely associated with *K. pneumoniae* and *Enterobacter* spp., mostly from clinical samples. The basal positioning of *mcr*‐9 and *mcr*‐10 suggests they may represent ancestral forms [56]. Among the host of *mcr*‐1, *mcr*‐2, and *mcr*‐5, *E. coli* showed the shortest branch, indicating a possible ancestral host of these variants [57]. *Aeromonas* displayed the shortest branches for *mcr*‐3, supporting an aquatic origin. *Moraxella* and *K. pneumoniae* emerged as likely ancestral hosts for *mcr*‐6 and *mcr-*7, whereas *R. ornithinolytica* and *E. cloacae* appeared to be probable origins for *mcr*‐8 and *mcr*‐10. For *mcr*‐4 and *mcr*‐9, similar branch lengths across *E. coli*, *Salmonella*, and *Enterobacter* indicate their emergence may have involved gene exchange within Enterobacteriaceae. In our study, *Aeromonas* and *K. pneumoniae* were the likely origins of *mcr-*3 and *mcr-*7, whereas *mcr-*1 and *mcr-*2 were attributed to *E. coli*. The discrepancies between our findings and those reported by Ling et al. [10] are primarily due to systematic differences in dataset composition rather than simple variation in sequence number. Ling et al. [10] analyzed approximately 70 sequences with a focus on early‐reported mcr variants (mainly *mcr*‐1 to *mcr*‐5), with stronger representation of animal‐associated isolates. In contrast, our study included 116 RefSeq‐curated nucleotide sequences covering all 10 recognized *mcr* variants (*mcr*‐1 to *mcr*‐10), with broader temporal coverage (2001–2024), wider host diversity (human, animal, and environmental sources), and expanded geographic representation. These differences in temporal, ecological, and taxonomic sampling likely influenced the inferred host–gene associations and phylogenetic clustering patterns observed in the two studies.

## 5. Limitations

We acknowledge that several limitations exist that should be considered when interpreting our findings. First, our literature search relied exclusively on the Scopus database. While Scopus offers excellent multidisciplinary coverage, this choice inevitably introduces data retrieval bias by omitting unique medical or regional publications indexed in PubMed or Web of Science. Additionally, restricting our dataset to English‐language peer‐reviewed articles means valuable local surveillance reports written in other languages were likely missed. Second, our author productivity metrics use a standard full counting method, which assigns equal weight to every coauthor. This tends to over‐represent researchers working within large, highly collaborative institutional networks without accounting for actual contribution or author order. Finally, uncorrected self‐citations may skew impact metrics, and this quantitative approach cannot assess the clinical or experimental validity of the underlying studies.

## 6. Conclusions

An integrated bibliometric, metadata, and phylogenetic analysis aimed at providing a comprehensive understanding of *mcr gene variants* in terms of distribution, diversity, and evolution was conducted. Our bibliometric analysis revealed that research on *mcr* has expanded rapidly since its first report in 2016. China and Germany emerged as major research and occurrence hotspots, reflecting their strong institutional capacity and intensive use of antibiotics in livestock production. It further highlighted the authors′ preference for a few high‐impact journals. Phylogenetic analysis showed distinct clustering of *mcr* variants, indicating independent evolutionary pathways. *mcr*‐1 exhibited the widest host and geographical range, primarily in *E. coli*, while *mcr*‐3 dominated aquatic environments, suggesting environmental reservoirs as critical transmission pathways. Branch length analysis suggested that *E. coli*, *Aeromonas*, and *Klebsiella* species were likely ancestral hosts of several *mcr* variants, reinforcing their role in AMR gene dissemination. Overall, the research landscape showed advances; however, key gaps remain. Surveillance‐based studies are limited in under‐represented regions, especially Africa, which bears one of the highest burdens of AMR and where weak genomic infrastructure restricts detection of *mcr* variants. Also unexplored are environmental reservoirs, including wastewater, soils, and aquatic ecosystems, despite their known role in the spread of AMR. In addition, the mechanisms behind coselection and codissemination of mcr genes with other resistance determinants, including carbapenemases, need to be understood. In the same vein, evolutionary drivers and costs of *mcr* variants are not understood within and outside clinical settings. In addition, the lack of standardized cross‐sectoral data integration limits the potential of One Health surveillance in addressing the mcr resistance menace. Given that *mcr*‐mediated resistance is globally distributed across diverse samples and hosts, future studies should prioritize longitudinal, multisectoral surveillance using genomics and metagenomics to improve detection and control. Thus, strengthening surveillance through a One Health approach that integrates metagenomics and/or genomic, ecological, and epidemiological data is crucial to mitigate the continued spread of colistin resistance genes.

## Author Contributions

M.Z.A. and U.O.E.: conception and design of the study. M.Z.A., U.O.E., U.C.T., F.N., and M.H.R.: acquisition of data. M.Z.A. and U.O.E.: analysis of data. M.Z.A. and U.O.E.: writing the original draft. M.H.R., E.A., H.M., and C.M.: review and editing. M.Z.A., M.H.R., E.A., H.M., and C.M.: screening the data for quality check. All authors have reviewed the manuscript and given approval for the publication.

## Funding

No funding was received for this manuscript.

## Disclosure

All the authors approved the final draft of the manuscript.

## Ethics Statement

This was not applicable in our study as there were no human or animal participants.

## Conflicts of Interest

The authors declare no conflicts of interest.

## Supporting Information

Additional supporting information can be found online in the Supporting Information section.

## Supporting information


**Supporting Information 1** Table S1: Accession numbers and metadata of the sequences.

**Supporting Information 2** Table S2: Number of published articles on *mcr* and citations per year from 2015 to 2025.

**Supporting Information 3** Table S3: Summary of the Top 30 journals that have published *mcr* studies and their metrics.

**Supporting Information 4** Table S4: Summary of the Top 20 most cited documents in *mcr*, the authors, DOIs, and total and average citations.

**Supporting Information 5** Table S5: Summary of the top most prolific authors with at least 30 *mcr*‐related articles.

**Supporting Information 6** Table S6: Bibliometric coupling metrics for the countries, including total link strength (TLS) calculated via VOSviewer.

**Supporting Information 7** Table S7: Top 10 leading funding sponsors in *mcr* research between 2015 and 2025.

## Data Availability

Data is available in the article′s supporting information.
