# Supplementary material for: Global Bibliometric and Phylogenetic Analysis of mcr‐Mediated Colistin Resistance
Source: Biomed Res Int. 2026 Jul 20;2026:8343626. doi: 10.1155/bmri/8343626 (PMC13382347; doi:10.1155/bmri/8343626)
Supplement: Supplementary file 1 — Supporting Information 1 Table S1: Accession numbers and metadata of the sequences. [file BMRI-2026-8343626-s001.docx]

**Supplementary Table 1.** Accession numbers and meta of the sequences

| Name | Date | Location |
| --- | --- | --- |
| NG_050417_E_coli_Swine_2019-11-09_mcr1 | 2019-11-09 | China |
| NG_055582_E_coli_Swine_POR_2015_mcr1 | 2015 | Portugal |
| NG_068218_E_coli_Human_GER_2018_mcr1 | 2018 | Germany |
| NG_051170_K_pneumoniae_Human_ITA_2014_mcr1 | 2014 | Ital |
| NG_064789_E_coli_CHI_2012_mcr1 | 2012 | China |
| NG_057460_K_pneumoniae_Chicken_CHI_2019-04-17_mcr1 | 2019-04-17 | China |
| NG_052861_E_coli_Chicken_CHI_2017-02-08_mcr1 | 2017-02-08 | China |
| NG_052893_S_enterica_Human_CHI_2014-05-03_mcr1 | 2014-05-03 | China |
| NG_055784_E_coli_Human_Peru_2016-10-03_mcr1 | 2016-03-03 | Peru |
| NG_061610_K_pneumoniae_Chicken_NA_Ref_seq_mcr1 | 2018-08-23 | NA |
| NG_068217_E_coli_Human_GER_2018_mcr1 | 2018 | Germany |
| NG_052663_E_coli_Human_ARG_2017_mcr1 | 2017 | Argentina |
| NG_052664_E_coli_NA_NA_mcr1 | 2017-01-25 | NA |
| NG_054678_E_coli_Sewage_CHI_2015-11-30_mcr1 | 2015-11-30 | China |
| NG_054697_E_coli_Poultry_Brunei_2017-05-01 | 2017-05-01 | Brunei |
| NG_057466_E_coli_Turkey_GER_212_mcr1 | 2012 | Germany |
| NG_065449_S_enterica_Egg_CHI_2024-04-08 | 2024-04-08 | Germany |
| NG_065451_E_coli_Human_CHI_2016_mcr1 | 2016 | China |
| NG_065944_E_coli_Chicken_NIG_2018-04-07_mcr1 | 2018-04-07 | Nigeria |
| NG_070762_E_coli_NA_NA_2020-09-18_mcr1 | 2020-09-18 | NA |
| NG_070763_uncultured_bacterium_ENV_CHI_2020-09-18_mcr1 | 2020-09-18 | China |
| NG_070764_uncultured_bacterium__ENV_CHI_2020-09-18_mcr1 | 2020-09-18 | China |
| NG_074755_E_coli_Human_ITA_2018_mcr1 | 2018 | Italy |
| NG_079262_E_coli_Urine_NA_2022-03-03_mcr1 | 2022-03-03 | NA |
| NG_079263_E_coli_Urine_NA_2022-03-03_mcr1 | 2022-03-03 | NA |
| NG_056412_E_coli_Swine_JAP_2015-06_mcr1 | 2015-06 | Japan |
| NG_064787_E_coli_Chicken_CHI_2019-04-27_mcr1 | 2019-04-27 | China |
| NG_064788_E_coli_Cattle_CHI_2019-04-27_mcr1 | 2019-04-27 | China |
| NG_065450_E_coli_Swine_UK_2019-06-26_mcr1 | 2019-06-26 | China |
| NG_074756_E_coli_Sputum_NA_2021-05-27_mcr1 | 2021-05-27 | UK |
| NG_203419_E_coli_Chicken_CHI_2023-07-11_mcr1 | 2023-07-11 | China |
| NG_231577_E_coli_Swine_GER_2016-11-25_mcr1 | 2016-11-25 | Germany |
| NG_242325_E_coli_Dog_ITA_2024-01-30_mcr1 | 2024-01-30 | Italy |
| NG_067237_E_coli_Poultry_BAN_2019-04-28_mcr1 | 2019-04-28 | Bangladesh |
| NG_067235_S_enterica_Poultry_BAN_2019-04-10_mcr1 | 2019-04-10 | Bangladesh |
| NG_067236_E_coli_Poultry_2019-03-07_mcr1 | 2019-03-07 | Bangladesh |
| NG_055583_Moraxella_sp_Swine_POR_2015_mcr1 | 2015-06 | Portugal |
| NG_051171_E_coli_NA_BEL_2018-01-19_mcr2 | 2018-01-19 | Belgium |
| NG_076679_E_coli_Swine_BEL_2015_mcr2 | 2015-06 | Belgium |
| NG_070766_uncultured_bacterium_ENV_CHI_2020-09-18_mcr2 | 2020-09-18 | China |
| NG_070768_uncultured_bacterium_ENV_CHI_2020-09-18_mcr2 | 2020-09-18 | China |
| NG_070767_uncultured_bacterium_ENV_CHI_2020-09-18_mcr2 | 2020-09-18 | China |
| NG_055496_M_pluranimalium_Swine_SPA_2001_mcr2 | 2001 | Spain |
| NG_065452_E_coli_Swine_THA_2016-09-09_mcr2 | 2016-09-09 | Thailand |
| NG_070765_uncultured_bacterium_ENV_Swine_2020-09-18_mcr2 | 2020-09-18 | China |
| NG_055781_Moraxella_sp_Swine_UK_2015_mcr6 | 2015 | UK |
| NG_055492_K_pneumoniae_Pus_THA_2015_mcr3 | 2015 | Thailand |
| NG_055493_K_pneumoniae_Urine_THA_2015_mcr3 | 2015 | Thailand |
| NG_055505_E_coli_NA_NA_2017-08-20_mcr3 | 2017-08-20 | NA |
| NG_055523_E_coli_THA_2016-08-11_mcr3 | 2016-08-11 | Thailand |
| NG_060583_K_pneumoniae_Human_2016-11-18_mcr3 | 2016-11-18 | Thailand |
| NG_056184_Escherichia_coli_NA_NA_2018-01-19_mcr3 | 2018-01-19 | NA |
| NG_070778_K_pneumoniae_Human_THA_2020-09-18_mcr3 | 2020-09-18 | Thailand |
| NG_064791_E_coli_Human_VIE_2015_mcr3 | 2015 | Vietnam |
| NG_070777_E_coli_Swine_THA_2020-09-18_mcr3 | 2020-09-18 | Thailand |
| NG_066546_K_pneumoniae_Stool_NA_2019-10-02_mcr3 | 2019-10-02 | NA |
| NG_065453_K_pneumoniae_Stool_NA_2019-06-26_mcr3 | 2019-06-26 | NA |
| NG_065455_K_pneumoniae_Stool_NA_2019-09-26_mcr3 | 2019-06-26 | NA |
| NG_055497_C_freundii_Swine_CHI_2014_mcr3 | 2014 | China |
| NG_055782_E_coli_NA_NA_2017-13-11_mcr3 | 2017 | NA |
| NG_060581_K_pneumoniae_Swine_THA_2016-08-11_mcr3 | 2016-08-11 | Thailand |
| NG_060580_E_coli_Human_THA_2015-03-08 | 2015-03-08 | Thailand |
| NG_055663_Aeromonas_hydrophila_Fish_GER_2017-11-14_mcr3 | 2017-11-14 | Germany |
| NG_055799_Aeromonas_caviae_Duck_NA_2017-11-14_mcr3 | 2017-11-14 | NA |
| NG_055661_Aeromonas_media_Turkey_GER_2018-02-14_mcr3 | 2018-02-14 | Germany |
| NG_057484_E_coli_Swine_BRA_2018-11-09_mcr3 | 2018-11-09 | Brazil |
| NG_055660_Aeromonas_allosaccharophila_Fish_GER_2018-01-02_mcr3 | 2018-01-02 | Germany |
| NG_055662_Aeromonas_hydrophila_Fish_GER_2017-11-14_mcr3 | 2017-11-14 | Germany |
| NG_070774_Aeromonas_jandaei_River_water_SOU_2020-01-30_mcr3 | 2020-01-30 | South_Africa |
| NG_070775_Aeromonas_veronii_River_water_SOU_2020-01-30_mcr3 | 2020-01-30 | South_Africa |
| NG_070772_Aeromonas_jandaei_River_water_SOU_2020-01-30_mcr3 | 2020-01-30 | South_Africa |
| NG_070773_Aeromonas_jandaei_River_water_SOU_2020-01-30_mcr3 | 2020-01-30 | South_Africa |
| NG_055783_Aeromonas_veronii_Chicken_CHI_2017-11-13_mcr3 | 2017-11-13 | China |
| NG_060585_Aeromonas_veronii_Human_CHI_2017-06_mcr3 | 2017 | China |
| NG_071230_Aeromonas_veronii_Blood_THA_2016-01-24_mcr3 | 2016-01-24 | Thailand |
| NG_060514_Aeromonas_caviae_Human_CHI_2017_mcr3 | 2017 | China |
| NG_060517_Aeromonas_salmonicida_Chicken_CHI_2017 | 2017 | China |
| NG_060519_Aeromonas_caviae_ENV_CHI_2017_mcr3 | 2017 | China |
| NG_060515_Aeromonas_bivalvium_ENV_CHI_2017_mcr3 | 2017 | China |
| NG_070769_uncultured_bacterium_Bats_CHI_2020-18-09_mcr3 | 2020-09-18 | China |
| NG_070770_uncultured_bacterium_Swine_CHI_2020-09-18_mcr3 | 2020-09-18 | China |
| NG_088452_Aeromonas_veronii_River_CHI_2022-10-06_mcr3 | 2022-10-06 | China |
| NG_060516_Aeromonas_media_Chicken_CHI_2017_mcr3 | 2017 | China |
| NG_064790_Aeromonas_hydrophila_Human_MAL_2017-04-27_mcr3 | 2017-04-27 | Malaysia |
| NG_070776_Aeromonas_caviae_Human_JAP_2014_mcr3 | 2014 | Japan |
| NG_070771_Aeromonas_jandaei_River_water_SOU_2020-01-30_mcr3 | 2020-01-30 | South_Africa |
| NG_060518_Aeromonas_allosaccharophila_Chicken_CHI_2017_mcr3 | 2017 | China |
| NG_088379_Aeromonas_veronii_Sewage_CHI_2019_mcr3 | 2019 | China |
| NG_056413_K_pneumoniae_Chicken_CHI_2018-02-14_mcr7 | 2018-02-14 | China |
| MK070339_E_coli_Human_FRA_2018-10-18_mcr9 | 2018-10-18 | France |
| MK791138_S_enterica_Human_USA_2010_mcr9 | 2010 | USA |
| MN049988_Enterobacter_cloacae_Human_CHI_2006-09-14_mcr9 | 2006-09-14 | China |
| NG_066767_Enterobacter_roggenkampii_Human_CHI_2019-10-25_mcr10 | 2019-09-14 | China |
| NG_079954_Enterobacter_cloacae_NA_NA_2022-03-31_mcr10 | 2022-03-31 | NA |
| NG_076638_Enterobacter_ludwigii_HUman_CHI_2011-10-23_mcr10 | 2011-10-23 | China |
| NG_076639_Enterobacter_kobei_Human_CHI_2012-12-03_mcr10 | 2012-12-03 | China |
| NG_076640_Enterobacter_kobei_Human_CHI_2011-09-23_mcr10 | 2011-09-23 | China |
| NG_057461_Enterobacter_cloacae_NA_NA_2018-05-24_mcr4 | 2018-05-24 | NA |
| NG_061608_S_enterica_NA_NA_23-08-2018_mcr4 | 2023-08-18 | NA |
| NG_231578_Swine_GER_2017-04-28_mcr4 | 2017-04-28 | Germany |
| NG_057464_E_coli_Swine_NA_2018-05-24_mcr4 | 2018-05-24 | NA |
| NG_057470_E_coli_Swine_GER_2011_mcr4 | 2011 | Germany |
| NG_057471_E_coli_Swine_GER_2011_mcr4 | 2011 | Germany |
| NG_057465_E_coli_Swine_NA_2018-05-24_mcr4 | 2018-05-24 | NA |
| NG_088453_Acinetobacter_baumannii_Papermill_FIN_2022-10-06_mcr4 | 2022-10-06 | Finland |
| NG_242769_Vibrio_cholerae_Bird_GER_2023-11-02 | 2023-11-02 | Germany |
| NG_061399_K_pneumoniae_Swine_CHI_2018-08-01_mcr8 | 2018-08-01 | China |
| NG_061627_K_pneumoniae_NA_NA_2018-08-23_mcr8 | 2018-08-23 | NA |
| NG_066547_K_pneumonia_Stool_NA_2019-10-02_mcr8 | 2019-10-02 | NA |
| NG_074757_Raoultella_ornithinolytica_Chicken_CHI_mcr8 | 2021-05-27 | China |
| NG_244661_uncultured_Klebsiella_ENV_NA_mcr8 | 2025-03-25 | NA |
| NG_055658_S_enterica _NA_GER_2011_mcr5 | 2011 | Germany |
| NG_065945_hospital_metagenome_Water_NER_23-06-2017_mcr5 | 2017-06-23 | Netherland |
| NG_057467_E_coli_Swine_GER_2011_mcr5 | 2011 | Germany |
| NG_061405_E_coli_Horse_BRA_2018-01-08_mcr5 | 2018-01-08 | Brazil |
| NG_231579_E_coli_Swine_GER_20-03-2006 | 2006-03-20 | Germany |
