# Supplementary material for: Global Bibliometric and Phylogenetic Analysis of mcr‐Mediated Colistin Resistance
Source: Biomed Res Int. 2026 Jul 20;2026:8343626. doi: 10.1155/bmri/8343626 (PMC13382347; doi:10.1155/bmri/8343626)
Supplement: Supplementary file 2 — Supporting Information 2 Table S2: Number of published articles on mcr and citations per year from 2015 to 2025. [file BMRI-2026-8343626-s002.docx]

**Supplementary Table 2.** Number published articles on *mcr* and citations per year from 2015 to 2025

| **Year** | **Publications (%)** | **Total citations** | **MeanTCperArt** | **MeanTCperYear** | **Citable Years** |
| --- | --- | --- | --- | --- | --- |
| 2015 | 1(0.03) | 331 | 331 | 30.09 | 11 |
| 2016 | 203(5.16) | 17484 | 86.13 | 8.61 | 10 |
| 2017 | 272(6.91) | 15197 | 55.87 | 6.21 | 9 |
| 2018 | 289(7.34) | 12320 | 42.63 | 5.33 | 8 |
| 2019 | 342(8.69) | 11625 | 33.99 | 4.86 | 7 |
| 2020 | 418(10.62) | 11345 | 27.14 | 4.52 | 6 |
| 2021 | 530(13.47) | 9715 | 18.33 | 3.67 | 5 |
| 2022 | 531(13.49) | 7068 | 13.31 | 3.33 | 4 |
| 2023 | 441(11.20) | 3947 | 8.95 | 2.98 | 3 |
| 2024 | 515(13.08) | 2019 | 3.92 | 1.96 | 2 |
| 2025 | 394(10.01) | 213 | 0.54 | 0.54 | 1 |

Note: MeanTCperArt= Mean total citations per article; MeanTCperYear= Mean total citations per year
