# Supplementary material for: Global Bibliometric and Phylogenetic Analysis of mcr‐Mediated Colistin Resistance
Source: Biomed Res Int. 2026 Jul 20;2026:8343626. doi: 10.1155/bmri/8343626 (PMC13382347; doi:10.1155/bmri/8343626)
Supplement: Supplementary file 3 — Supporting Information 3 Table S3: Summary of the Top 30 journals that have published mcr studies and their metrics. [file BMRI-2026-8343626-s003.docx]

**Supplementary Table 3.** Summary of the top 30 journals that have published *mcr* studies and their metrics

| **Journals** | **Articles (%)** | **Citations** | **IF (2024)** | **Quartile ranking** |
| --- | --- | --- | --- | --- |
| Frontiers in Microbiology | 350(8.89) | 7873 | 4.5 | Q1 |
| Journal of Global Antimicrobial Resistance | 204(5.18) | 2001 | 3.2 | Q2 |
| Antimicrobial Agents and Chemotherapy | 188(4.78) | 8563 | 4.5 | Q1 |
| Journal of Antimicrobial Chemotherapy | 174(4.42) | 1898 | 3.6 | Q2 |
| Antibiotics | 174(4.42) | 7334 | 4.6 | Q1 |
| Microbiology Spectrum | 152(3.86) | 2164 | 3.8 | Q2 |
| Microbial Drug Resistance | 136(3.46) | 2234 | 1.9 | Q3 |
| International Journal of Antimicrobial Agents | 130(3.30) | 3585 | 4.6 | Q1 |
| Infection and Drug Resistance | 105(2.67) | 1665 | 2.9 | Q2 |
| Frontiers in Cellular and Infection Microbiology | 68(1.73) | 742 | 4.8 | Q2 |
| Science of the Total Environment | 61(1.55) | 1272 | 8.0 | Q1 |
| Plos One | 50(1.27) | 996 | 2.6 | Q2 |
| Microorganisms | 47(1.19) | 1608 | 4.2 | Q2 |
| International Journal of Food Microbiology | 43(1.09) | 732 | 5.2 | Q1 |
| Scientific Reports | 41(1.04) | 899 | 3.9 | Q1 |
| mSphere | 41(1.04) | 1094 | 3.1 | Q2 |
| BMC Microbiology | 37(0.94) | 415 | 4.2 | Q2 |
| Journal of Medical Microbiology | 37(0.94) | 516 | 2.0 | Q3 |
| Veterinary Microbiology | 36(0.91) | 435 | 2.7 | Q3 |
| Microbial Genomics | 36(0.91) | 586 | 4.0 | Q1 |
| Lancet Infectious Diseases | 31(0.79) | 7771 | 31.0 | Q1 |
| mBio | 30(0.76) | 2500 | 4.7 | Q1 |
| Pathogens | 30(0.76) | 297 | 3.3 | Q2 |
| Total | 2,525 (64.15) |  |  |  |

IF = Impact factor; Q1, 2 and 3 = First, second and third quartiles.
