# Supplementary material for: Global Bibliometric and Phylogenetic Analysis of mcr‐Mediated Colistin Resistance
Source: Biomed Res Int. 2026 Jul 20;2026:8343626. doi: 10.1155/bmri/8343626 (PMC13382347; doi:10.1155/bmri/8343626)
Supplement: Supplementary file 4 — Supporting Information 4 Table S4: Summary of the Top 20 most cited documents in mcr, the authors, DOIs, and total and average citations. [file BMRI-2026-8343626-s004.docx]

**Supplementary Table 4.** Summary of the most top 20 most cited documents in *mcr*, the authors, dois, total and average citations

| Rank | **Articles** | **DOI** | **Total citations** | **Total citations per year** |
| --- | --- | --- | --- | --- |
| 1 | Liu YY, 2016, Lancet Infect. Dis. | 10.1016/S1473-3099(15)00424-7 | 4445 | 445 |
| 2 | Xavier BB, 2016, Eurosurveillance | 10.2807/1560-7917.ES.2016.21.27.30280 | 673 | 67 |
| 3 | Poirel L, 2018, Microbiol. Spectr. | 10.1128/microbiolspec.ARBA-0026-2017 | 643 | 80 |
| 4 | Bevan ER, 2017, J. Antimicrob. Chemother. | 10.1093/jac/dkx146 | 612 | 68 |
| 5 | Rebelo AR, 2018, Eurosurveillance | 10.2807/1560-7917.ES.2018.23.6.17-00672 | 552 | 69 |
| 6 | Yin W, 2017, Mbio | 10.1128/mBio.00543-17 | 535 | 59 |
| 7 | Wang R, 2018, Nat. Commun. | 10.1038/s41467-018-03205-z | 509 | 64 |
| 8 | Zhang R, 2017, Ebiomedicine | 10.1016/j.ebiom.2017.04.032 | 479 | 53 |
| 9 | Borowiak M, 2017, J. Antimicrob. Chemother. | 10.1093/jac/dkx327 | 447 | 50 |
| 10 | Carattoli A, 2017, Eurosurveillance | 10.2807/1560-7917.ES.2017.22.31.30589 | 443 | 49 |
| 11 | Wang X, 2018, Emerg. Microbes Infect. | 10.1038/s41426-018-0124-z | 418 | 52 |
| 12 | Carroll LM, 2019, Mbio | 10.1128/mBio.00853-19 | 415 | 59 |
| 13 | Sun J, 2019, Nat. Microbiol. | 10.1038/s41564-019-0496-4 | 391 | 56 |
| 14 | Wang C, 2020, Emerg. Microbes Infect. | 10.1080/22221751.2020.1732231 | 373 | 62 |
| 15 | Baron SA, 2016, Int. J. Antimicrob. Agents | 10.1016/j.ijantimicag.2016.06.023 | 333 | 33 |
| 16 | Trimble MJ, 2016, Cold Spring Harb. Perspect. Med. | 10.1101/cshperspect.a025288 | 333 | 33 |
| 17 | Hasman H, 2015, Eurosurveillance | 10.2807/1560-7917.ES.2015.20.49.30085 | 331 | 30 |
| 18 | McGann PT, 2016, Antimicrob. Agents Chemother. | 10.1128/AAC.01103-16 | 330 | 33 |
| 19 | Jeannot KK, 2017, Int. J. Antimicrob. Agents | 10.1016/j.ijantimicag.2016.11.029 | 308 | 34 |
| 20 | Yang Y, 2018, J. Antimicrob. Chemother.-A | 10.1093/jac/dky111 | 304 | 38 |

DOI = Digital online identifier
