# Supplementary material for: Global Bibliometric and Phylogenetic Analysis of mcr‐Mediated Colistin Resistance
Source: Biomed Res Int. 2026 Jul 20;2026:8343626. doi: 10.1155/bmri/8343626 (PMC13382347; doi:10.1155/bmri/8343626)
Supplement: Supplementary file 5 — Supporting Information 5 Table S5: Summary of the top most prolific authors with at least 30 mcr‐related articles. [file BMRI-2026-8343626-s005.docx]

**Supplementary Table 5.** Summary of the top most prolific authors with at least 30 *mcr*-related articles

| **Rank** | **Authors** | **Articles (%)** | **Citations** | **Institution** | **Country** |
| --- | --- | --- | --- | --- | --- |
| 1 | Ruichao Li | 77(1.96) | 1583 | Yangzhou University | China |
| 2 | Wang Y | 62(1.58) | 7803 | China Agricultural University | China |
| 3 | Wang Z | 55(1.40) | 1001 | Yangzhou University | China |
| 4 | Poirel L | 49(1.24) | 3113 | University of Fribourg | Switzerland |
| 5 | Zhang R | 49(1.24) | 7071 | Zhejiang University | China |
| 6 | Patrice N | 48(1.22) | 3007 | University of Fribourg | Switzerland |
| 7 | Sun J | 45(1.14) | 1873 | South China Agricultural University | China |
| 8 | Chen S | 44(1.12) | 1545 | The Hong Kong Polytechnic University | Hong Kong |
| 9 | Liu J | 37(0.94) | 1161 | South China Agricultural University | China |
| 10 | Rolain JM | 37(0.94) | 1447 | Aix Marseille University | France |
| 11 | Chan EWC | 36(0.91) | 1352 | Hong Kong Polytechnic University | Hong Kong |
| 12 | Li Y | 36(0.91) | 755 | South China Agricultural University | China |
| 13 | Liao X | 33(0.84) | 915 | South China Agricultural University | China |
| 14 | Xu H | 33(0.84) | 728 | South China Agricultural University | China |
| 15 | Feng Y | 32(0.81) | 2050 | Zhejiang University | China |
| 16 | Liu Y | 32(0.81) | 782 | South China Agricultural University | China |
| 17 | Wang J | 31(0.79) | 399 | South China Agricultural University | China |

**Note:** Author publication counts were determined using the standard full counting method via VOSviewer, where a single multi-authored publication is credited as one full unit to every contributing researcher. Consequently, individual productivity values may be inflated within collaborative networks and do not account for author order, specific role designation, or varying contribution weights. Ranking distributions should be interpreted with caution.
