# Supplementary material for: Global Bibliometric and Phylogenetic Analysis of mcr‐Mediated Colistin Resistance
Source: Biomed Res Int. 2026 Jul 20;2026:8343626. doi: 10.1155/bmri/8343626 (PMC13382347; doi:10.1155/bmri/8343626)
Supplement: Supplementary file 6 — Supporting Information 6 Table S6: Bibliometric coupling metrics for the countries, including total link strength (TLS) calculated via VOSviewer. [file BMRI-2026-8343626-s006.docx]

Supplementary Table 6: Bibliometric coupling metrics for the countries, including Total Link Strength (TLS) calculated via VOSviewer.

| ID | Country | Documents | Citations | Total link strength |
| --- | --- | --- | --- | --- |
| 1 | China | 1434 | 35200 | 491860 |
| 2 | United States | 466 | 19680 | 239516 |
| 3 | United Kingdom | 306 | 15585 | 155125 |
| 4 | France | 215 | 7596 | 112511 |
| 5 | Brazil | 179 | 2586 | 74337 |
| 6 | Germany | 179 | 6758 | 83106 |
| 7 | India | 174 | 2568 | 53203 |
| 8 | Japan | 174 | 2797 | 87986 |
| 9 | Spain | 155 | 3956 | 55501 |
| 10 | Italy | 151 | 4110 | 74773 |
| 11 | Egypt | 125 | 2517 | 59177 |
| 12 | Switzerland | 116 | 4890 | 77812 |
| 13 | Thailand | 106 | 1419 | 57144 |
| 14 | Australia | 100 | 2922 | 55010 |
| 15 | South Korea | 80 | 1088 | 43371 |
| 16 | Hong Kong | 77 | 2661 | 45240 |
| 17 | Canada | 75 | 2669 | 36135 |
| 18 | Netherlands | 75 | 2148 | 40951 |
| 19 | Portugal | 67 | 1502 | 37171 |
| 20 | Pakistan | 66 | 1306 | 27411 |
| 21 | Sweden | 60 | 1851 | 30002 |
| 22 | Denmark | 59 | 2632 | 29491 |
| 23 | Iran | 57 | 839 | 17498 |
| 24 | Saudi Arabia | 53 | 946 | 25493 |
| 25 | Turkey | 52 | 592 | 25437 |
| 26 | Viet Nam | 52 | 1088 | 27957 |
| 27 | Belgium | 50 | 2990 | 37140 |
| 28 | Czech Republic | 50 | 1287 | 23208 |
| 29 | Argentina | 47 | 783 | 24080 |
| 30 | Taiwan | 47 | 640 | 15758 |
| 31 | South Africa | 45 | 1083 | 18654 |
| 32 | Austria | 42 | 879 | 16242 |
| 33 | Greece | 41 | 590 | 13598 |
| 34 | Mexico | 39 | 610 | 13519 |
| 35 | Lebanon | 37 | 644 | 17792 |
| 36 | Bangladesh | 35 | 420 | 13468 |
| 37 | Tunisia | 34 | 753 | 8752 |
| 38 | Ireland | 30 | 480 | 15340 |
| 39 | United Arab Emirates | 29 | 490 | 9787 |
| 40 | Algeria | 28 | 557 | 11406 |
| 41 | Croatia | 28 | 382 | 6341 |
| 42 | Norway | 28 | 458 | 15249 |
| 43 | Poland | 28 | 385 | 11947 |
| 44 | Singapore | 27 | 691 | 10481 |
| 45 | Chile | 22 | 362 | 7594 |
| 46 | Nigeria | 22 | 531 | 4943 |
| 47 | Ecuador | 21 | 306 | 10150 |
| 48 | Malaysia | 20 | 246 | 6587 |
| 49 | Nepal | 19 | 465 | 11799 |
| 50 | Russian Federation | 19 | 268 | 7675 |
| 51 | Colombia | 16 | 406 | 4411 |
| 52 | Iraq | 16 | 125 | 3727 |
| 53 | Israel | 16 | 433 | 5507 |
| 54 | Finland | 15 | 351 | 4393 |
| 55 | Hungary | 13 | 429 | 3375 |
| 56 | Peru | 13 | 149 | 6957 |
| 57 | Uruguay | 12 | 106 | 2674 |
| 58 | Romania | 11 | 143 | 3860 |
| 59 | Bulgaria | 10 | 76 | 4264 |
| 60 | Morocco | 10 | 58 | 3019 |
| 61 | Ghana | 9 | 210 | 1971 |
| 62 | Philippines | 9 | 160 | 3319 |
| 63 | Qatar | 9 | 135 | 4740 |
| 64 | Tanzania | 9 | 101 | 1005 |
| 65 | Kenya | 8 | 88 | 1436 |
| 66 | Kuwait | 8 | 181 | 3097 |
| 67 | Nevis | 7 | 1031 | 6648 |
| 68 | Serbia | 7 | 96 | 2737 |
| 69 | New Zealand | 6 | 260 | 2375 |
| 70 | Sudan | 6 | 116 | 2145 |
| 71 | Myanmar | 5 | 86 | 1412 |
| 72 | Uganda | 5 | 41 | 406 |
