# Supplementary material for: Global Bibliometric and Phylogenetic Analysis of mcr‐Mediated Colistin Resistance
Source: Biomed Res Int. 2026 Jul 20;2026:8343626. doi: 10.1155/bmri/8343626 (PMC13382347; doi:10.1155/bmri/8343626)
Supplement: Supplementary file 7 — Supporting Information 7 Table S7: Top 10 leading funding sponsors in mcr research between 2015 and 2025. [file BMRI-2026-8343626-s007.docx]

**Supplementary Table 7.** Top 10 leading funding sponsors in *mcr* research between 2015 and 2025

| **Rank** | **Funding Sponsor** | **Country** | **Documents (%)** |
| --- | --- | --- | --- |
| 1 | National Natural Science Foundation of China | China | 835(21.21) |
| 2 | National Key Research and Development Program of China | China | 299(7.60) |
| 3 | Conselho Nacional de Desenvolvimento Científico e Tecnológico | Brazil | 105(2.67) |
| 4 | National Institutes of Health | USA | 101(2.57) |
| 5 | Coordenação de Aperfeiçoamento de Pessoal de Nível Superior | Brazil | 93(2.36) |
| 6 | Priority Academic Program Development of Jiangsu Higher Education Institutions | China | 92(2.34) |
| 7 | Japan Society for the Promotion of Science | Japan | 89(2.26) |
| 8 | European Regional Development Fund | European Union | 88(2.24) |
| 9 | Natural Science Foundation of Zhejiang Province | China | 85(2.16) |
| 10 | National Institute of Allergy and Infectious Diseases | USA | 84(2.13) |
